# Internet Interpersonal Connection Mediates the Association between Personality and Internet Addiction

**DOI:** 10.3390/ijerph16193537

**Published:** 2019-09-21

**Authors:** Yun-Hsuan Chang, Yun-Ting Lee, Shulan Hsieh

**Affiliations:** 1 Department of Psychology, College of Medical and Health Science, Asia University, Taichung 41354, Taiwan; 2 Department of Medical Research, China Medical University Hospital, China Medical University, Taichung 40447, Taiwan; 3 Department of Psychiatry, College of Medicine, National Cheng Kung University, Tainan 701, Taiwan; 4 Department of Psychology, National Cheng Kung University, Tainan 701, Taiwan; nf3jcxkd@yahoo.com.tw; 5 Institute of Allied Health Sciences; National Cheng Kung University, Tainan 701, Taiwan; 6 Department and Institute of Public Health, National Cheng Kung University, Tainan 701, Taiwan

**Keywords:** Internet addiction, Internet interpersonal interaction, neuroticism, personality, vulnerability

## Abstract

Backgrounds: The development of the Internet has changed interpersonal interactions, so that people no longer need to physically meet each other. However, some people are more vulnerable to becoming addicted to Internet activities, something to which the ease of Internet access and usage has contributed. In this study, we examined the association between personality traits and feelings about online interpersonal interactions to predict Internet addiction. This was accomplished using an online advertisement that asked participants to complete the questionnaires in the laboratory. Methods: Two hundred and twenty-three participants with a mean age of 22.50 years were recruited for this study and asked to complete the following questionnaires: the Beck Depressive Inventory (BDI), the Beck Anxiety Inventory (BAI), the Chen Internet Addiction Scale (CIAS), the Eysenck Personality Questionnaire (EPQ), the Internet Usage Questionnaire (IUQ) and the Feelings of Internet Interpersonal Interaction Questionnaire (FIIIQ). Results: The results showed that people with a neurotic personality and anxious feelings about Internet interpersonal interactions are more likely to become addicted to the Internet. In addition, people with neuroticism and who are more anxious about Internet interpersonal relationships are more likely to develop Internet addiction. Conclusions: People who tend to develop new interpersonal relationships via the Internet and be anxious about online interpersonal relationships are more vulnerable to becoming addicted to the Internet. The individuals who are more anxious about Internet interpersonal interaction and tend to develop new interpersonal relationships via the Internet are more likely to develop Internet addiction.

## 1. Introduction

The Internet was developed in the early 1970s [1] and has gradually become much more common and popular. However, problems with Internet addiction (IA) have started to raise concerns, and IA has been introduced as a new clinical disorder [2,3]. Previous studies have indicated that maladaptive patterns of Internet use constitute addictive behavior, and Internet use on school campuses and in society in general has increased in recent years. Moreover, an etiopathogenetic model was suggested for the effect of unbalanced emotional regulation in developing internet addiction [4]. Although the Internet plays an important role in interpersonal communication and connections, as well as receiving family and social support [5], its extensive use could cause negative psychological effects, like for example, loss of self-control in using the Internet and becoming isolated in relationships [6,7,8,9]. 

### 1.1. The Attachment Type and IA

Nowadays, Internet use is a popular form of interpersonal connection which does not require face-to-face interactions which seems to help individuals maintain their current relationships and develop new ones without having to meet people in real life. The ease of accessibility of the Internet, however, has changed interpersonal interactions and increases the probabilities of developing Internet Addiction (IA). So far, a number of studies have been conducted to explore what factors may be related to developing Internet addiction. The attachment type is one of the possible factors that has been reported for developing online interpersonal relationships and IA [10,11,12]. Eichenberg et al. [11] conducted an online survey and assessed the correlation between attachment style, IA symptoms, and online relationships. A positive correlation between insecure attachment style and tendency for pathological Internet use, regardless of being web- or app-based, was reported. Individuals with ambivalent attachment were found to have more significant motives for anonymity and were more likely to seek emotional support than those with secure attachment. In addition, using the Rorschach inkblot test, researchers found that individuals with problematic Internet use showed lower social interaction. Even though people differ on the definition of pathological Internet use (PIU) [13], they agree that no matter the definition it includes and increases the probabilities to develop Internet Addiction and anxiety, which are both related to emotional dysregulation [14]. On the other hand, some people who have difficulty talking to others may prefer to communicate via the Internet; this is referred to as a social compensatory function. In addition, those referred to as escapists may use the Internet to escape from their real life, for example, when they fail in their academic performance or have problems in interpersonal interactions, they escape from their real life by using the Internet. Escapism and the social compensatory function have been found to be associated with pathological Internet use and insecure attachment style [11]. While ambivalent attachment users may want social interaction, they have difficulty accepting or opening up to others [15]. 

Moreover, because of the anonymity and emotional support from the Internet, people with anxious attachment have been reported to be more likely to become cyber addicts [16]. It may be that the Internet provides a platform for individuals who have anxious attachment and are more likely to seek reassurance from their online connections [7,16,17,18]. Moreover, for some people, excessive use of the internet may represent a compensatory strategy to cope with unpleasant feelings of anxiety, depression and loneliness [19].

Anxious attachment individuals with insecure attachment in their relationships with other people often seek reassurance, and the Internet and social media help to maintain their social network [20]. People with anxiety and insecure attachment have been found to have more interactions online and to be more involved in Internet social connections, while people with secure attachment have a lower tendency to become Internet addicts [15]. However, inconsistent results have been reported regarding the effect of attachment style on problematic Internet use; instead, it has been found that the male gender and depressive symptoms were predictive of IA [21]. The frequency and patterns of using the Internet as an instrument of interpersonal interactions might not be only correlated with attachment style. Lin et al. [15] found similar results to previous reports that anxious attached people have a higher tendency to become cyber addicts, and further found that the association between anxious attachment and IA was partially mediated by Internet interpersonal interaction. They also stated that there may be more than one type of attachment within an individual which influences interpersonal interaction. Based on this, they developed a questionnaire, the Feelings of Internet Interpersonal Interaction Questionnaire (FIIIQ), in Chinese with three types of feelings: close, anxiety, and dependence. They found no significant correlation between secured attachment and any dimensions of feelings of Internet interpersonal interaction but found a significant correlation between anxious attachment and dependence and anxious feelings [15]. 

### 1.2. Mood State and IA

IA has been defined as excessive behavior regarding Internet use which leads to impairment or distress [2]. Depression has been reported to be an important factor for Internet addicts [8,22,23,24,25]. Kim et al. [8] reported that a higher rate of depression was found in the IA group compared with those without IA in adolescents. Papastylianou et al. [22] investigated the correlation between the Big Five personality traits (neuroticism, openness, conscientiousness, extraversion and agreeableness) and depression regarding IA. They recruited students from the social sciences, humanities, and exact sciences departments and asked them to complete self-reported questionnaires of IA, personality traits (NEO-FFI), and depression. The results showed that an open personality was significantly associated with IA, and neuroticism was marginally associated with IA, which became weaker when controlling for depression. The results imply that the probability of an adolescent becoming addicted to the Internet increases when they are depressed. In addition, Bahrainian et al. [24] reported similar findings of predictable variables of depression and self-esteem for IA in university students. Moreover, Yucens and Uzer [23] had similar findings, finding that undergraduate medical students with IA had significantly higher scores on the Beck Depression Inventory (BDI). Whether depression is a possible risk factor for IA, a consequence of IA or a combination of both remains unclear. However, a clear correlation between depression and IA has been supported. 

### 1.3. Personality and IA

Another important factor of IA concerns personality traits. Previous studies have reported a positive relationship between extroversion and social media use [26,27] and addictive tendencies towards the Internet [28]. Another commonly studied personality trait, neuroticism, has also been reported to be positively associated with IA [29,30], specifically, social media use [20,26]. Extroverted people have been reported to play fewer online games and to be less likely to treat Internet interpersonal interactions and offline social networks equally. However, people with neuroticism are more likely to utilize the Internet to increase their self-esteem by developing a sense of group belonging. Moreover, a study with meta-analysis of association between Big Five personality traits and IA showed a strong correlation between neurotic personality trait and IA, but a negative association between extraversion and IA. 

Reviewing previous studies, the attachment style may be a factor between personality and IA. Based on previous studies about attachment, individuals with anxious or avoidant attachment to their parents may affect their interaction with other people, for example friends [31] or even romantic relationship, intimacy relationship or their spouse when they grow up [32]. The Internet has become a new method for people to develop new relationships nowadays. According to Lin’s findings [15], one type of attachment might be able to explain the association between attachment and the tendency towards IA. They suggested that feelings of online interpersonal interaction may be more suitable for studying the relationship between personality traits and IA. People with anxiety or dependent attachment were reported to have a higher desire for Internet interpersonal interaction. According to this idea, and results from previous findings of the relationship between neurotic personality and IA, the hypothesis in the current study is that how an individual feels about online interpersonal interaction may be connected to personality traits and IA. 

## 2. Methods

This study was approved by the Institutional Review Board (IRB, B-ER-101-144) of the National Cheng Kung University. All participants signed an informed consent form before participating in the study. The purpose of this study was to (1) compare different domains of personality traits, moods, and feelings regarding Internet activities between IA and non-IA groups; (2) investigate how people's feelings about various Internet activities correlate with different activities for Internet use; (3) explore the influences of personality traits on IA; (4) investigate the association between internet interpersonal connection and IA; (5) compare Internet activities between IA and non-IA individuals; (6) and investigate the mediating role of anxious feelings in the association between neuroticism and IA. 

### 2.1. Participants

Participants aged 20–30 years who were defined as digital natives [33] were recruited via an online advertisement. In total, 233 participants (mean age: 22.5 ± 2.07 years; 116 males and 117 females), normal or corrected-to-normal vision and who had no history of neurological, psychiatric or cardiovascular diseases in their self-report were recruited and required to complete all the questionnaires. 

### 2.2. Procedure

Each participant was first required to complete the Beck Depression Inventory (BDI) [34,35,36] and the Beck Anxiety Inventory (BAI) [37,38,39,40] to screen out those who possibly had mood disorders, such as depressive or anxiety disorders. The following questionnaires were then given to the participants to complete: Chen’s Internet Addiction Scale (CIAS-R) [41], Internet Usage Questionnaire (IUQ) [42], Eysenck Personality Questionnaire (EPQ) [43,44,45], Feelings of Internet Interpersonal Interaction Questionnaire (FIIIQ), and the Goal of Internet Interpersonal Interaction (GIII) [15]. 

### 2.3. Measurements

In the first version, neuroticism–stability and extroversion–introversion dimensions were suggested by the Eysenck personality theory and the EPQ was then established [44]. The extroversion dimension represents sociality and impulsivity. Individuals in this dimension are defined as enjoying social interactions, being energetic, and preferring social situations to loneliness. The neuroticism dimension is hypothesized as emotional instability and reactiveness, and that individuals who score high on this dimension tend to be anxious, depressive, overly emotional, shy, and have low self-esteem.

### 2.4. Goal of Internet Interpersonal Interaction

Three types of Internet interpersonal interactions are considered in the GIII index: maintaining current interpersonal relationships (TMCIPR), developing new interpersonal relationships (TDNIPR), and the degree of involvement in social networks (DISN) [15].

### 2.5. Feelings of Internet Interpersonal Interaction Questionnaire

The FIIIQ was established and modified according to Collins’ working model of adult attachment and interpersonal relationships [46]. There are three subscales of the FIIIQ: close, anxiety, and dependence, in which high scores in the anxiety and dependence subscales have been reported to be associated with ambivalent attachment, and low scores in the subscale of close is related to avoidance attachment [15].

### 2.6. Statistical Analysis

Pearson’s *r* correlation analysis was conducted to explore the associations among the variables. SPSS 22.0 (SPSS Inc., Chicago, IL, USA) was used for statistical analysis. To further explore the mediating role of personality in the association between feelings of Internet interpersonal interaction and IA, bootstrap analysis was carried out. In addition, structural equation modeling (SEM) was employed to empirically test the hypothesis that anxious feelings play a partial mediating role between the neuroticism personality trait, developing new social relationships, and IA. The fit index was computed to evaluate model fit with RMSEA < 0.06 [47,48].

## 3. Results

A total of 233 participants with a mean age of 22.5 years (SD = 2.07 years old), ranging from 20 to 30 years old, were recruited from an Internet advertisement. Of these, 116 were males, with a mean age of 22.59 ± 1.90 years, and 117 were females, with a mean age of 22.41 ± 2.24 years. All of the participants received at least 12 years of education; 75.5% of participants were university students and 24.0% were postgraduate students. None of the participants were at risk of depressive or anxiety disorders (BDI = 4.30 ± 3.76 (< 13), BAI = 1.91 ± 2.07 (<7)). 

In order to compare Internet interpersonal interaction, IA and personality between participants with and without IA, with a cut-off score of 64 or more in the CIAS-R-R [49], were used to identify those who may have IA. This diagnostic cut-off value of 63/64 has been demonstrated to have a high sensitivity rate (86.6%) and a diagnostic accuracy rate of 87.6%. This discriminative potential makes the scale a reliable diagnostic tool in an epidemiological survey, as it can provide the estimated prevalence rate and identify the target case group. Using this cut-off point score, the participants were divided into two groups: non-IA and IA. No significant difference of gender and educational level was found between the two groups (χ^2^ = 1.35, *p* = 0.25; χ^2^ = 3.28, *p* = 0.35), respectively. In order to investigate whether the two groups differed, a multivariate analysis of variance (MANOVA) was conducted with diagnostic group as the independent variable and all other variables as the dependent variables. The results were significant (F(10, 23122) = 24.0137.95, *p* < 0.0005). Univariates showed no significances for BDI score, all subscales of CIAS-R, anxious feelings and neuroticism scales, close Internet interpersonal relationship feelings, and extroverted personality between the two groups (*p* < 0.05) (Table 1). 

Pearson correlation analysis showed a significantly positive correlation between FIIIQ and GIII; for addiction, all the subscales of FIIIQ were positively associated with all the subscales of the III Index, except for TMCIPR (Table 2). Individuals with anxiety and dependence feelings on Internet activities had higher scores on the domain of developing new interpersonal interactions and their involvement in social networks, but this was not the case for those having closed feelings. Individuals with closed feelings were more likely to develop new interpersonal interactions but not to become significantly involved in social networks (Table 2). 

To further explore the association among IA tendencies, Pearson correlation analyses were conducted to explore the relationship among CIAS-R score, subscales of CIAS-R, subdomains of FIIIQ, and different personality traits. The results showed a significantly positive correlation among CIAS-R score, subscales of CIAS-R and neuroticism, and anxious feelings on the FIIIQ (Table 2). 

**Figure 1:**
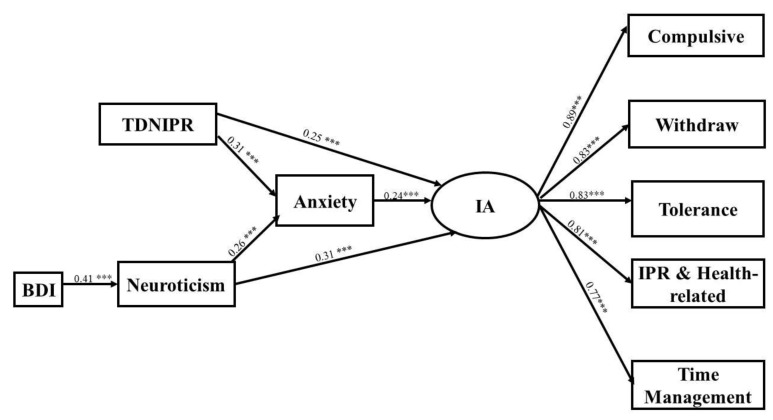
The model of the significant path coefficients between variables. *** *p* < 0.001.

To test the hypothesized model, path analysis was applied. The IA dimensions of CIAS-R were included in the model as latent variables, and the unidimensional constructs of neuroticism, anxious feelings about Internet interpersonal interaction, and BDI were included as observable variables. Goodness of fit indices of the model (Figure 1) indicated good fit to the data, and the model was acceptable (χ^2^/df = 1.30, RMSEA = 0.036, SRMR = 0.04, CFI = 0.99). 

The bootstrapping analysis showed that TDNIPR (β = 0.07, *p* < 0.001; 95% CI (0.09, 0.41)) and neuroticism (β = 0.06, *p* < 0.001; 95% CI (0.19, 0.43)) were indirectly associated with IA via anxious feelings about Internet interaction. Anxious feelings about Internet interpersonal interaction partially mediated neuroticism and TDNIPR with IA. Moreover, BDI was only associated with neuroticism (β = 0.41, *p* < 0.001), and there was no direct relationship between BDI (β = 0.13, *p* > 0.05) and IA. Neuroticism was found to fully mediate BDI with IA (Table 3). 

## 4. Discussion

The results of the current study showed significantly higher scores on depression, neuroticism, and anxious feelings about Internet interaction in the IA group than in the non-IA group. This is in line with previous findings on the relationship between depression and IA [8, 23–25]. Although there were more males in the IA group, no significant difference for gender was found. 

Moreover, neuroticism was found to be a mediator for depression with IA, which supported a previous finding that more depressive symptoms, positive outcome expectancy, and insecure attachment were found among Internet addicts [50]. The degree of depression could be worth considering in future work to explore the correlation with neuroticism and IA [22]. The association between depression and IA was fully mediated by neuroticism, which might explain a previous report that individuals with borderline personality disorder (BPD) are more neurotic and use the Internet as a platform to reduce their tension [51].

Because the Internet has become a popular platform for individuals to maintain and develop relationships, people interact online more easily nowadays than before. For some people who seek interpersonal relationships but fear being socially excluded, the Internet may be useful for developing interpersonal relationships without face-to-face interaction. There have been a number of studies that have examined the relationship between personality or attachment and IA. Anxious attachment has been reported to be an important factor in becoming an Internet addict, and secured attached individuals also show a higher tendency of involvement in Internet interpersonal interactions [15]. However, secured attached people are less likely to become Internet addicts compared with anxious attached people. This study adapted the hypothesis of attachment types to feelings of Internet interaction and found that individuals with anxious feelings about Internet interpersonal interaction are more likely to become Internet addicts. This finding is consistent with previous research showing that people with anxious and insecure attachment tend to exhibit problematic Internet use and become addicted to the Internet [12,15,26]. In addition, according to Lin et al.’s [15] findings that individuals may have combined attachment types, the subscales, anxiety, and dependence scores of FIIIQ were added up as the feelings of Internet interpersonal interaction (FIII) score, which has been suggested to be a mediator between anxious attachment and IA. A significantly positive correlation was found between FIII and CIAS-R, which supports a previous study which found that anxious and dependence feelings might be two factors that influence people with anxious attachment to become Internet addicts [15]. 

Moreover, neuroticism was also found here to correlate with the tendency of IA, which is consistent with previous findings [18,20,27,30,52,53]. Neuroticism was found to correlate with all the subdomains of cyber addiction, including the core symptoms of compulsion, withdrawal, and tolerance as well as other relative symptoms of interpersonal relationships, health-related problems, and time management. In addition, the relationship between neuroticism and IA was found to be mediated by anxious feelings. This finding may be used for the prevention of cyber addiction, in which Internet activities with less social interaction would reduce the probability of becoming addicted to the Internet for those with neuroticism and those who easily become anxious about social activities. 

We did not find the correlation between extroversive personality and any domains of IA in the current study, because we did not categorize IA into more specific categories, such as online gamers and social media use. Previous findings determined that extroverted individuals tend to be addicted to social media use [26,52,53,54]. It could be that most of the Internet activities the participants engaged in were online games. Playing online games is not like social media use, by which people can exhibit themselves and broadcast their preferences to communicate with others. Online game playing is done more likely to promote players’ game-winning skills rather than to establish interpersonal interactions. Online game players have been suggested to be shy and more introverted [55]. The results of the current study showed a negative tendency between extroversion and all domains of IA, although it did not reach statistical significance. Participants who were engaged in playing the MMORPG “World of Warcraft” online game were examined and a negative correlation was found between their playing time and the extroversion personality type [18], which was consistent with a previous suggestion that extraverts prefer to have face-to-face interpersonal interactions and use the Internet in a more instrumental and goal-oriented manner [56]. Neurotic individuals were found to tend to utilize the Internet for social interaction to help them feel a sense of belonging to a social network when they felt lonely, although they did not engage in communication-related activities such as group discussions or net meetings [56]. 

There are a few limitations in the current study. One limitation was that no specific online games were categorized to compare Internet interaction among online games, since more and more online games are developed and combined with social media use. In addition, our data were limited to self-report measurements, the validity of which is contingent on the accuracy of their reports. Furthermore, the sample size was relatively small, and a larger sample may be needed. 

Future research should continue to examine the factors related to why participants are anxious about Internet interpersonal relationships. In addition, individuals high in neuroticism may have a great amount of anxiety about interpersonal relationships, and the Internet may be a useful tool since numerous types of Internet uses may have different effects on individuals’ Internet usage. 

## 5. Conclusions

Feelings about Internet interpersonal interaction mediate individuals with a neurotic personality and the degree of IA. People who are more neurotic and anxious about developing interpersonal relationships through the Internet are more vulnerable to becoming addicted to Internet interaction. 

## Figures and Tables

**Table 1:** Comparisons between groups with Internet addiction (IA) and non-IA.

Variables	Non-Internet Addiction (*n* = 157)		Internet Addiction (*n* = 76)		Statistics (*p*)
	Mean	SD	Mean	SD	
Age	22.34	2.08	22.83	2.04	2.82 (0.94)
Gender (Male/Female)	74/83		42/34		1.35 (0.25)
BDI	3.90	3.59	5.12	3.98	5.51 (0.20)
BAI	1.78	2.06	2.20	2.09	2.12 (0.15)
CIAS-R					
Compulsive	9.77	2.36	14.36	1.90	217.50 (<0.0005)
Withdraw	10.96	2.40	14.91	1.93	156.89 (<0.0005)
Tolerance	8.44	1.73	11.78	1.80	185.55 (<0.0005)
Interpersonal Relationship and Health-Related Problems	12.69	2.86	18.51	2.79	215.08 (<0.0005)
Time Management	9.14	2.38	14.05	2.72	197.88 (<0.0005)
FIIIQ					
Close	15.04	2.41	15.46	2.34	1.60 (0.21)
Anxiety	13.54	3.52	15.91	3.55	23.15 (<0.0005)
Dependence	14.60	3.22	15.75	3.68	5.95 (0.015)
EPQ					
Neuroticism	3.73	2.59	5.14	2.77	14.71 (<0.0005)
Extroversion	9.85	3.40	8.99	4.17	2.82 (0.10)
GIII Index					
To maintain their current interpersonal relationships	4.36	.84	4.55	0.66	2.97 (0.09)
To develop new interpersonal relationships	4.96	1.48	6.05	1.70	25.50 (<0.0005)
Involvement of social networks	2.93	1.08	3.38	.78	10.58 (0.001)
Time spent on Internet activities (hours/week)					
game playing	5.42	10.19	11.08	13.93	12.31 (0.001)
social media	10.41	14.46	12.78	13.40	1.43 (0.03)
browsing and forums	20.92	22.23	29.11	34.04	1.43 (0.23)
others	6.34	7.09	8.00	7.27	2.75 (0.10)

* *p*-value < 0.05; ** *p*-value < 0.001; *** *p*-value < 0.0005.

**Table 2:** The correlational matrix for Internet addiction score, personality trait score, and Internet interpersonal interaction scores.

Questionnaires and Scales	Mean	SD	1	2	3	4	5	6	7	8	9	10	11	12	13	14	15	16	17	18	19
1. BDI	4.3	3.76	1	0.55 ***	0.08	0.07	0.10	−0.01	−0.04	−0.09	0.09	−0.11	0.22 **	0.42 ***	−0.06	0.24 ***	0.21**	0.26***	0.25 ***	0.15 *	0.19 *
2. BAI	1.91	2.07		1	0.03	0.03	0.15	−0.03	−0.01	0.18 **	0.07	0.003	0.19 *	0.34 ***	−0.04	0.10	0.07	0.10	0.09	0.05	0.12
3. GIII	14.31	3.69			1	0.47 ***	0.87 ***	0.67 ***	0.34***	0.21 **	0.29 ***	0.24 ***	0.27 ***	0.09	0.24 ***	0.38 ***	0.30 ***	0.38 ***	0.34 ***	0.28 ***	0.37 ***
4. TMCIPR	9.57	3.68				1	0.20 **	0.05	−0.07	0.05	−0.14 *	−0.02	0.14 *	0.01	0.15 *	0.09	0.03	0.11	0.11	0.03	0.14 *
5. TDNIPR	44.46	7.13					1	0.37 **	0.26 ***	0.09	0.29 ***	0.16 *	0.28 ***	0.05	0.17 *	0.31 ***	0.26 ***	0.35 ***	0.27 ***	0.23 ***	0.24 ***
6. DISN	15.18	2.39						1	0.28 ***	0.02	0.38 ***	0.15 *	0.19	0.05	0.17 *	0.31 ***	0.26 ***	0.35 ***	0.27 ***	0. 23 ***	0.24 ***
7. FIIIQ	44.46	3.94							1	0.66 ***	0.76 ***	0.16 **	0.19 *	0.06	0.17 *	0.28 ***	0.23 ***	0.25 ***	0.26 ***	0.23 ***	0.25 ***
8. Close	14.97	2.72								1	0.21 **	0.45 ***	0.08	0.25 ***	0.07	0.02	0.01	−0.01	0.03	0.02	0.07
9. Anxiety	15.18	3.4									1	0.35 ***	0.25 ***	0.29 ***	0.07	0.38 ***	0.37 ***	0.36 ***	0.32 ***	0.32 ***	0.29 ***
10. Dependence	14.31	4.1										1	0.07	−0.11	0.15 *	0.15 *	0.08	0.14 *	0.17 *	0.13	0.16 *
11. EPQ	13.76	4.1											1	0.48 ***	0.76 ***	0.22 **	0.18 *	0.18 *	0.23 ***	0.23 ***	0.23 ***
12. Neurot.	4.19	2.72												1	−0.21 *	0.38 ***	0.22 *	0.18 *	0.18 ***	0.23 ***	0.23 ***
13. Extrov.	9.57	3.68													1	−0.04	−0.07	−0.05	−0.03	−0.08	0.05
14. CIAS–R score	58.38	13.59														1	0.90 ***	0.85 ***	0.85 ***	0.88 ***	0.85 ***
15. Com.	11.27	3.09															1	0.77 ***	0.73 ***	0.72 ***	0.67 ***
16. Withd.	12.25	2.92																1	0.69 ***	0.65 ***	0.62 ***
17. Toler.	9.53	2.35																	1	0.68 ***	0.64 ***
18.IPR and Health	14.59	3.94																		1	0.64 ***
19. TM	10.74	3.4																			1

BDI: Beck Depression Inventory; BAI: Beck Anxiety Inventory; CIAS-R: Chen’s Internet Addiction Scale; Com.: compulsion subscale of CIAS-R; Withd.: Withdraw subscale of CIAS-R; IPR and Health: Interpersonal relationship and health-related problems subscale of CIAS-R; TM: Time management subscale of CIAS-R; EPQ: Eysenck Personality Questionnaire; Extrov.: Extroversion; Neurot.: Neuroticisim; GIII: Goal of Internet Interpersonal Interaction; TMCIPR: To maintain current interpersonal relationship; TDNIPR: To development new interpersonal relationship; DISN: degree of involvement in social networks; FIIIQ: Feelings of Internet Interpersonal Interaction Questionnaire; FIII: Feelings of Internet Interpersonal Interaction; * *p*-value < 0.05; ** *p*-value < 0.001; *** *p*-value < 0.0005.

**Table 3:** Standardized estimates of total, direct, and indirect effects on IA and mediator variables.

Association between Variables	Estimate	Effect	SE	% Explained of Total Effect
TDNIPR → IA (total effect)	0.53	0.25	0.10	
TDNIPR → IA (direct effect)	0.41	0.18	0.05	72
TDNIPR → Anxious feelings →IA (indirect effect)	0.12	0.07	0.01	28
Neuroticism → IA (total effect)	0.38	0.31	0.06	
Neuroticism → IA (direct effect)	0.32	0.25	0.08	81
Neuroticism → Anxious feelings →IA (indirect effect)	0.06	0.06	0.048	19
BDI → Neuroticism → IA	0.30	0.13	0.04	

TDNIRP: to develop new interpersonal relationship, the index of Internet interpersonal interaction.
